# Repurposing the bacterial surface display technology for drug delivery

**DOI:** 10.1016/j.addr.2025.115701

**Published:** 2025-10-03

**Authors:** Shaobo Yang, Mengdi Yang, Maria Jennings, Hania Timek, Amber E. Haley, Rizwan Romee, Jiahe Li

**Affiliations:** a Department of Medical Oncology, Dana-Farber Cancer Institute, Boston, MA 02215, United States; b Department of Bioengineering, Northeastern University, Boston, MA 02115, United States; c Department of Biomedical Engineering, University of Michigan, Ann Arbor, MI 48109, United States

**Keywords:** Bacterial surface display, Synthetic biology, Drug delivery, Microbiome

## Abstract

Bacteria have emerged as versatile platforms for therapeutic delivery, owing to their inherent adaptability, genetic tractability, and ability to interface with the human microbiome and immune system. This review explores the evolution of bacterial engineering for medical applications, emphasizing drug delivery strategies enabled by bacterial surface display technologies. We outline the advantages of surface display, such as enhanced localization, prolonged therapeutic activity, and reduced systemic toxicity, over conventional bacterial secretion and lysis-based delivery methods. The review details key biological mechanisms of surface display in both Gram-negative and Gram-positive bacteria, including outer membrane proteins, sortase-mediated anchoring, and spore-based systems. We also highlight emerging applications of surface-displayed cytokines, nanobodies, and immunomodulatory proteins in cancer therapy, vaccine development, microbiome engineering, and animal health. Innovative approaches combining bacterial display with conjugation systems and biosensors expand the potential of these living therapeutics for precise, responsive, and programmable interventions. Furthermore, we propose a future roadmap that leverages computational tools such as AlphaFold and in silico screening to rationally identify optimal outer membrane anchors, accelerating the design of next-generation surface display platforms. While challenges remain, including regulatory hurdles and microbial stability, continued interdisciplinary innovation with synthetic biology promises to transform engineered bacteria into clinically viable therapeutic agents. This review positions bacterial surface display as a powerful and underexplored modality for targeted drug delivery, bridging synthetic biology, immune engineering, and translational medicine.

## GRAPHICAL ABSTRACT

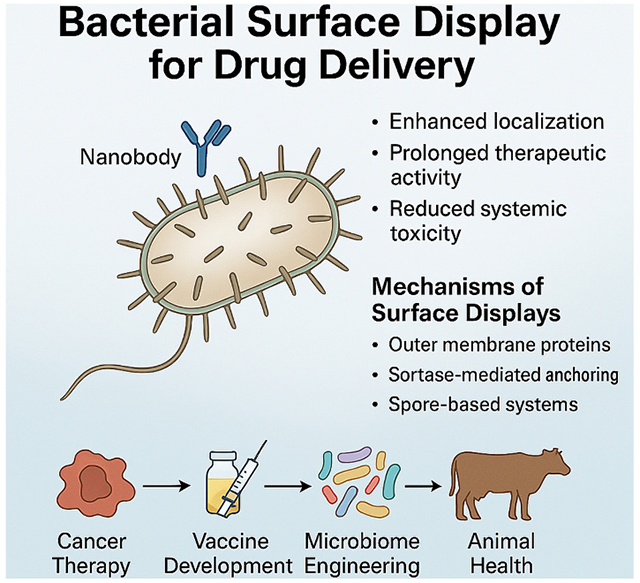

## 1. The relationships between humans and microbes

**Fig. 1.**
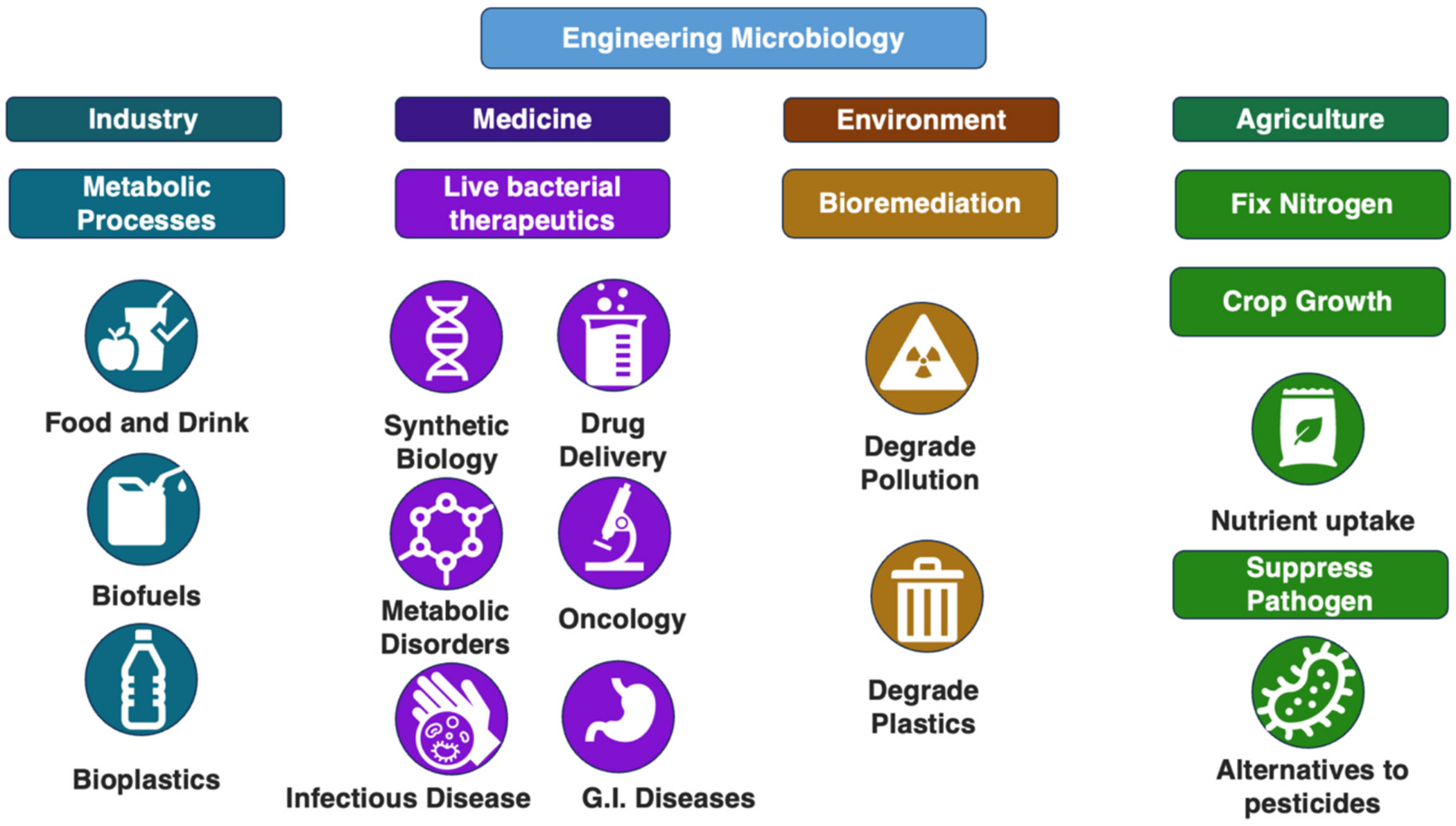
Microbiology engineering techniques are increasingly being used across different fields of technology. However, this review focuses on the current and developing applications of engineered microbiology in drug delivery. Genetically engineered live bacteria are becoming an increasingly popular therapeutic modality to treat metabolic disorders, cancers, infectious diseases, and gastrointestinal diseases. The cartoons were generated using BioRender.

The relationship between humans and microbiology is profoundly interwoven, with microbial organisms playing crucial roles in health, disease, and environmental adaptation. The human microbiome, comprising trillions of microorganisms that colonize the skin [1], gastrointestinal tract [2], respiratory system [3], oral cavities [4], and other body sites, profoundly influences physiological processes such as digestion, immune modulation, and even neurodevelopment. These microbes engage in complex interactions with their human host, contributing to the metabolism of dietary components, synthesis of essential vitamins, and protection against pathogenic invasion. However, imbalances in microbial communities, or dysbiosis, have been implicated in numerous diseases, including metabolic disorders [5], autoimmune conditions [6], and cancers [7,8]. Advances in microbiology have highlighted not only the diverse roles of microbes in maintaining homeostasis but also their potential as therapeutic targets and vectors for treatment. The study of microbial-host interactions has thus emerged as a critical field, where understanding the underlying mechanisms of these relationships could unlock novel strategies to harness or modulate microbial functions for improved health outcomes (Fig. 1) [9–13].

Besides, humans have harnessed microbiology in numerous ways, from health and medicine to agriculture, industry, and environmental management [14–18]. In medicine, beneficial microbes are employed as probiotics to support gut health [15], while genetically engineered bacteria produce essential biopharmaceuticals, such as insulin and growth hormones, for treating diseases [18]. Advances in synthetic biology have also enabled the development of bacterial vectors for targeted drug delivery, gene therapy, and cancer immunotherapy [14,17,18]. In agriculture, microbial inoculants enhance crop growth by improving nutrient uptake, fixing nitrogen, and suppressing pathogens, offering a sustainable alternative to chemical fertilizers and pesticides [17]. Industrial microbiology capitalizes on microbial metabolism for processes like fermentation, which produces food and beverages, and biofuels and bioplastics that contribute to greener energy and materials [14,19]. Furthermore, microbes play a critical role in environmental biotechnology, where they are utilized in bioremediation to degrade pollutants and restore contaminated ecosystems [16].

## 2. Engineering bacteria for therapeutic interventions

The engineering of bacteria for therapeutic applications has evolved remarkably since its inception, becoming a pivotal strategy in medicine, agriculture, and environmental remediation. This innovative field leverages the inherent genetic and metabolic capabilities of bacterial organisms to develop therapeutic agents, encompassing probiotics, vaccine delivery systems, and biopharmaceuticals. The groundwork for bacterial engineering was laid in the 1970s with the discovery of plasmids, which enabled the manipulation of bacterial genetics. This landmark achievement facilitated the introduction of foreign genes into bacterial hosts, culminating in the production of recombinant proteins such as insulin and human growth hormone, transforming the landscape of therapeutic interventions for various diseases [20,21].

As advancements in molecular biology and genetic engineering progressed, researchers began to explore the potential of bacteria as living therapeutics [22]. A notable development in this realm was using bacteria as a robust platform to produce biopharmaceuticals, among which engineering bacteria as anti-cancer therapeutics represents an innovative frontier in oncology, harnessing bacterial specificity, adaptability, and genetic manipulability to target tumors with precision. By modifying naturally tumor-colonizing strains, such as *Escherichia coli* (*E. coli*), *Salmonella enterica* (*S. enterica*), and *Clostridium novyi* (*C. novyi*), researchers have created bacterial strains capable of selectively homing to tumor sites, particularly to hypoxic and necrotic regions that are challenging for traditional therapies to access [22–29]. Once localized within the tumor microenvironment, these engineered bacteria can deliver a range of anti-cancer agents, such as cytotoxic proteins, prodrug-converting enzymes, or immune-stimulatory molecules, directly to the tumor, maximizing local therapeutic effect while sparing healthy tissues [30–37].

Recent advancements in synthetic biology have enabled bacteria design with programmed genetic circuits that allow precise control over therapeutic delivery in response to environmental cues within the tumor, such as low oxygen levels, quorum sensing, or acidic pH [38–41]. For instance, probiotic *E. coli* strain Nissle 1917 (*EcN*) engineered to express STING agonist cyclic di-adenylate (*c*-di-AMP) under hypoxic conditions has demonstrated significant tumor suppression in preclinical syngeneic murine colorectal and melanoma models [38]. Besides, integrating a self-lysis circuit in *EcN* achieved synchronized delivery of immunomodulating molecules for enhanced anti-cancer therapy in various preclinical mouse models, including melanoma, colorectal, and lung cancers [39–45]. Clinically, *Clostridium novyi-NT*, an anaerobic strain, has shown promise in early clinical trials by selectively lysing tumor cells and activating immune responses within solid tumors [46]. These bacterial therapy approaches hold considerable promise in overcoming the limitations of conventional treatments, offering a highly targeted, programmable, and adaptable platform for cancer therapy with potential applications in personalized medicine.

In parallel, engineering bacteria to manipulate the gut microbiome holds vast potential for therapeutic applications, particularly in treating gastrointestinal diseases, metabolic disorders, and immune-related conditions. Recent studies have demonstrated the efficacy of engineered probiotics and commensal strains, such as *Lactococcus lactis, EcN*, commensal *E. coli EcAZ*, and *Bacteroides* species, which have been genetically modified to produce anti-inflammatory biologics or inhibit pathogenic species [47–51]. For instance, *Lactococcus lactis* has been engineered to produce interleukin-10 (IL-10), an anti-inflammatory cytokine, directly in the gut, helping to alleviate symptoms of inflammatory bowel disease (IBD) in animal models [52].

Furthermore, synthetic biology has enabled the creation of “smart” bacterial systems equipped with gene circuits that can sense and respond to environmental cues, such as pH, temperature, and specific biomolecules. These engineered systems include synthetic biosensors integrated into bacterial genomes to detect biomarkers associated with inflammation, infection, and cancer. For example, *E. coli* strains were engineered with synthetic circuits that detect nitric oxide (NO), a marker of inflammation, and then release anti-inflammatory agents or antibiotics in response. This approach has been explored to treat gastrointestinal infections and reduce inflammation in mouse models [53–61].

The CRISPR-Cas system has also been leveraged to engineer precise bacterial genome edits, allowing for sophisticated control over microbial functions. The innovative approaches involve engineering bacteriophages or Horizontal Gene Transfer (HGT) machines equipped with CRISPR-Cas components to selectively target and remove antibiotic resistance genes or pathogenic bacteria in the gut microbiome, which could be a critical tool in addressing the rise of antibiotic-resistant infections [62–65].

## 3. Therapeutic delivery strategies to engineer bacteria for disease treatment

In the following section, we outline different modes of therapeutic bacterial delivery systems, including but not limited to bacterial secretion, bacterial surface display, and bacterial lysis [66–71]. While this review focuses on the bacterial surface display for drug delivery and therapeutic applications, it is helpful first to discuss bacterial secretion systems since understanding the biological mechanisms underlying protein secretion is key to appreciating the complexity and advantages of the bacterial surface display. Traditional bacterial therapies typically rely on bacterial secretion or self-lysis to deliver therapeutic agents. Bacterial secretion or self-lysis involve the continuous production and release of therapeutic molecules such as cytokines, enzymes, and antimicrobial peptides directly at the target site. Despite their potential, secretion-based and self-lysis-based delivery approaches that rely on the production of soluble therapeutic proteins frequently suffer from rapid clearance from the target site. Once secreted into the extracellular space, soluble proteins can diffuse away from the site of interest, becoming diluted in interstitial fluids and entering systemic circulation, thereby reducing their local concentration within the target site, such astumor microenvironment (TME), limiting their efficacy and therapeutic window [72–74].

In contrast, bacterial surface display offers a more controlled and stable delivery approach. By presenting proteins or peptides directly on the bacterial cell surface, this method allows prolonged interaction with target cells and enhanced therapeutic efficacy. Therefore, after exploring bacterial secretion mechanisms, we will discuss bacterial surface display as an alternative promising strategy for developing therapeutic and vaccine carriers. Additionally, toward the end of this section, we will briefly discuss the bacterial lysis-based approach, which leverages genetically engineered bacteria to accumulate therapeutic payloads in cytosol released upon cell lysis.

Notably, in addition to the individual advantages offered by surface display, secretion, or lysis-based systems, recent studies suggest that combining these approaches can further enhance therapeutic delivery. Specifically, integrating surface display with secretion mechanisms, conjugative plasmid transfer, or programmed cell lysis can enable both targeted and sustained delivery of therapeutic payloads. For instance, surface-anchored nanobodies can facilitate specific cell targeting, while concurrent secretion systems (e.g., T3SS or T4SS) inject therapeutic proteins into host or bacterial recipient cells. Similarly, conjugation-based systems can transfer plasmids encoding toxic or regulatory genes in a controlled fashion, particularly when donor-recipient binding is enhanced via surface display elements such as nanobody-antigen pairs [75,76]. Finally, engineered lysis circuits enable temporal control of intracellular cargo release upon colonization of disease-relevant niches [68]. Such combinatorial strategies expand the functionality and precision of bacterial therapeutics, supporting the development of next-generation drug delivery systems that are programmable, responsive, and safe.

### 3.1. Secretion

One primary mode of delivery is protein secretion, where bacteria are engineered to release therapeutic proteins and peptides directly at the target site. For instance, as discussed earlier, *L. lactis* has been modified to secrete IL-10 to treat IBD by reducing inflammation within the gut [52]. Additionally, clinical trials have explored the use of L. *lactis* strains that secrete human Trefoil Factor 1 to treat oral mucositis [77] and strains secreting proinsulin and IL-10 for type 1 diabetes (NCT03751007) [78].

**Fig. 2.**
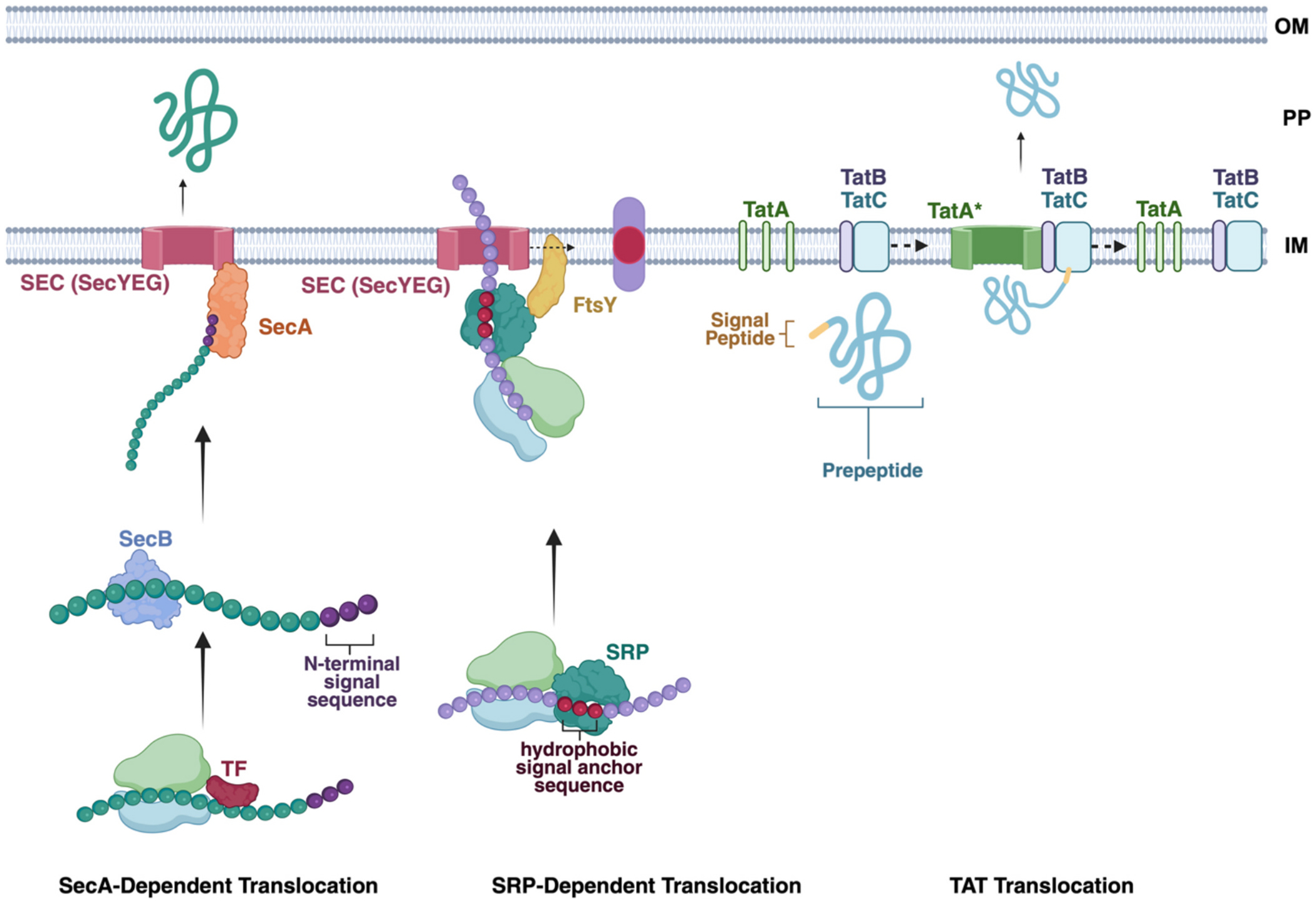
Translocation from cytoplasm to periplasmic space by SEC and TAT translocation systems. SEC translocation is broadly characterized by two pathways, a SecA-dependent pathway and SRP-dependent pathway. The SecA-dependent pathway most commonly acts post-translation, and the SRP-dependent pathway typically works co-translationally, while there are exceptions to both [87–89]. The TAT system, in contrast, exports fully folded proteins that require cytoplasmic assembly or cofactor binding prior to translocation. OM: outer membrane; PP: periplasm; IM: inner membrane.

Bacterial secretion systems mediate the transport of proteins across the cytoplasmic and outer membranes and into the extracellular milieu or directly into host cells. These pathways are critical for bacterial physiology, virulence, and biotechnological applications. The Sec and Tat systems are the primary means by which proteins are translocated across the inner membrane in Gram-negative and membrane in Gram-positive bacteria (Fig. 2).

The Sec pathway is the most evolutionarily conserved and the primary route for exporting proteins in an unfolded state across the cytoplasmic membrane in both Gram-negative and Gram-positive bacteria. It comprises the SecYEG translocon, an integral membrane protein complex, and the peripheral ATPase SecA [79,80]. The mechanism of protein secretion based on the Sec pathway can be divided into four parts. First, proteins destined for secretion possess an N-terminal signal peptide that directs them to the Sec machinery. In the post-translational pathway, the chaperone SecB maintains the preprotein in an unfolded conformation and delivers it to SecA. Alternatively, in the co-translational pathway, the signal recognition particle (SRP) recognizes the emerging signal peptide and targets the ribosome-nascent chain complex to the SecYEG translocon [79–81]. Then, SecA binds to both the preprotein and the SecYEG channel, inserting a two-helix finger (THF) into the channel’s cytoplasmic funnel. This interaction positions the preprotein for translocation [82–84]. Afterwards, SecA undergoes cycles of ATP binding and hydrolysis, inducing conformational changes that drive segments of the preprotein through the SecYEG channel. The THF acts as a piston, pushing the polypeptide into the channel during ATP binding and retracting upon hydrolysis, while a clamp domain prevents backsliding of the translocating chain. The process continues reiteratively only for a few cycles, after which SecA disengages and ATP-dependent translocation is replaced by the proton motive force [82]. Lastly, once translocation is complete, the signal peptide is cleaved by signal peptidase, and the mature protein folds into its functional conformation in the periplasm or is further directed to its destination, outer membrane or extracellular export via systems like T2SS or T5SS [82,85,86].

The twin-arginine translocation (Tat) pathway specializes in transporting fully folded proteins across the cytoplasmic membrane, a process essential for proteins that acquire cofactors or fold in the cytoplasm. The Tat system comprises three core components: TatA, TatB, and TatC [79,90–92]. Similarly, the mechanism of protein secretion based on the Tat pathway can be divided into four parts. At the beginning, substrate proteins contain an N-terminal signal peptide with a conserved twin-arginine (RR) motif. This motif is recognized by the TatBC receptor complex in the membrane. Secondly, upon substrate binding, TatB is displaced, allowing TatA to be recruited to the complex. TatA oligomerizes to form a transient pore through which the folded protein can pass. Then, the translocation of the folded protein is driven by the proton motive force (PMF) across the membrane. The exact mechanism by which PMF facilitates this process is still under investigation, but it is known that ATP is not required for Tat-dependent translocation. Finally, after translocation, the signal peptide is cleaved, and the mature protein is released into the periplasm. The Tat components then disassemble and are recycled for subsequent rounds of translocation [79,80].

**Fig. 3.**
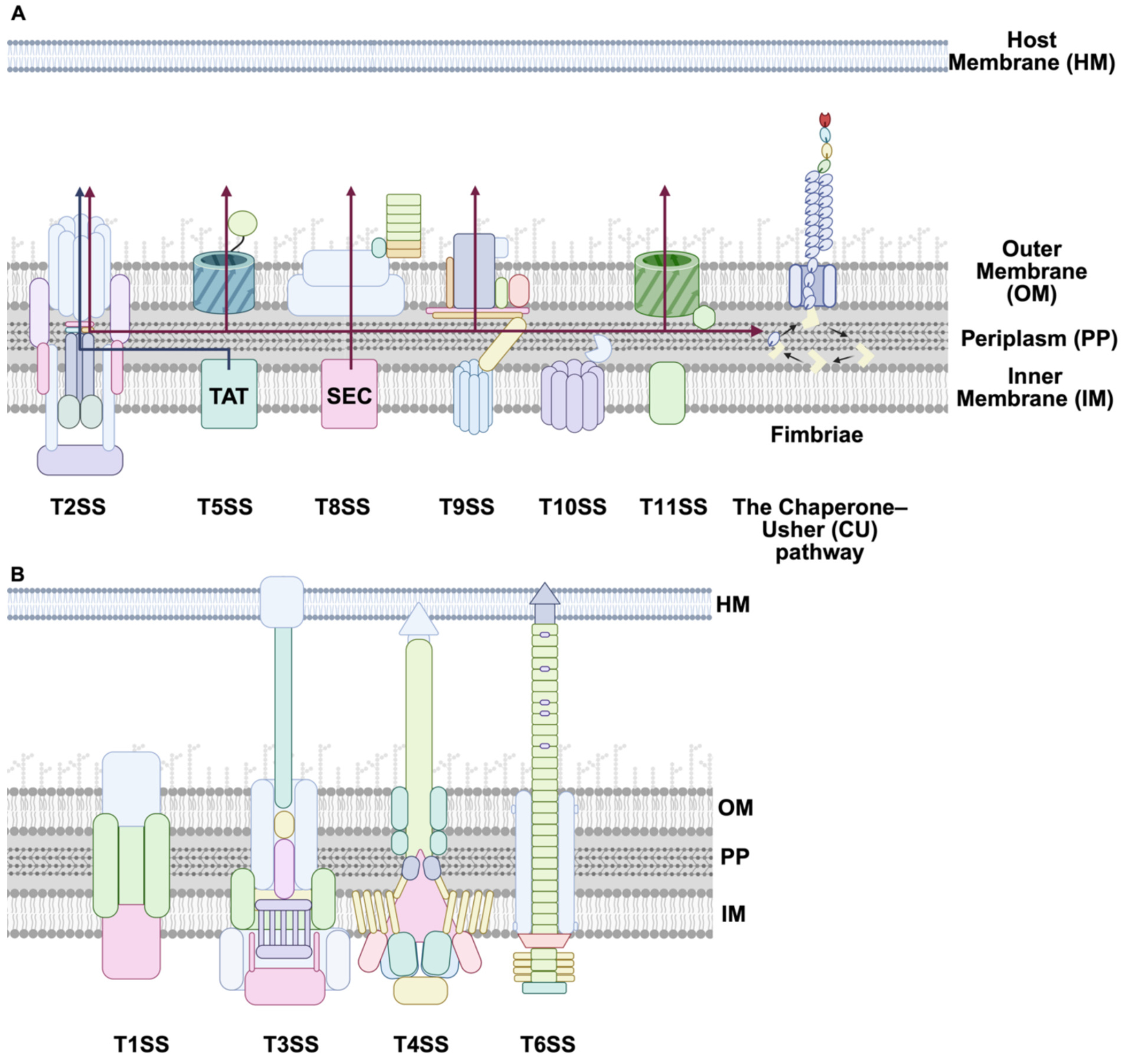
The secretion systems presented in the upper panel **(A)** translocate substrates via a two-step translocation pathway that rely on the Tat or Sec pathways for delivery across the IM and the dedicated pathway for delivery across the OM. **(B)** The lower panel delivers proteins in one step from the bacterial cytoplasm to the cell surface, either for delivery of proteins to the milieu or into target cells [93,111]. Specifically, Type I secretion system (T1SS) allows direct export to the extracellular space using an ATP-binding cassette transporter. T2SS transports proteins from the periplasms to extracellular space, while T3SS employs-like structure to inject straight into host cells. T4SS can transport DNA to the host cell, which is important for virulence and horizontal gene transfer [85–87,89,92,96,97,99,103,107,108,112–122]. The cartoons were generated using BioRender.

Beyond the inner membrane, Gram-negative bacteria utilize a broad array of dedicated secretion systems (T1SS–T11SS) and chaperone-usher pathway, while Gram-positive bacteria use adapted forms of these systems despite lacking an outer membrane [85,93]. Below is a summary of the best-characterized secretion systems and their functions (Fig. 3):

T1SS (Type I): A Sec-independent system that directly translocates substrates from the cytoplasm to the extracellular space via a tripartite complex comprising an ABC transporter, a membrane fusion protein, and an outer membrane protein (e.g., HlyB/HlyD/TolC) [85,94].

T2SS (Type II): Transports folded proteins from the periplasm (delivered via Sec or Tat) to the extracellular environment. It uses a piston-like pseudo pilus to push substrates through a secretin pore [95].

T3SS (Type III): A syringe-like injectisome that directly delivers effector proteins into host cells. Found in many pathogens such as *Salmonella* and *Shigella*, it is essential for manipulating host cellular processes [75,96].

T4SS (Type IV): Transports both DNA and protein substrates into host cells or other bacteria, playing roles in conjugation and virulence. Examples include *Agrobacterium tumefaciens* and *Helicobacter pylori* [97–99].

T5SS (Type V): Proteins are first transported to the periplasm via the Sec pathway and then auto-translocate across the OM using their own β-barrel domain (Type Va), or with a separate translocator in two-partner systems (Type Vb) [86,100,101].

T6SS (Type VI): A contractile nanomachine structurally similar to phage tails, delivering toxic effectors into prokaryotic or eukaryotic cells. T6SS is crucial in bacterial competition and pathogenesis [102].

T7SS (Type VII): First characterized in *Mycobacterium tuberculosis*, this system exports WXG100-family proteins through the thick mycolic acid-rich outer membrane of *Actinobacteria* [103].

T8SS (Type VIII): Associated with curli fiber biogenesis in *E. coli*. It mediates the export and polymerization of amyloid-like proteins involved in adhesion and biofilm formation [104].

T9SS (Type IX): Present in *Bacteroidetes*, this system secretes and anchors enzymes and adhesins involved in gliding motility and proteolysis [105].

T10SS (Type X): A specialized Sec-independent system that exports bacteriocins and colicins. These are often toxins used to outcompete other microbes [85].

T11SS (Type XI): Recently identified, less well-characterized systems. T11SS may relate to outer membrane vesicle-mediated delivery [106].

Chaperone-usher pathway: The chaperone–usher (CU) pathway is a specialized protein secretion system in Gram-negative bacteria responsible for assembling adhesive surface structures known as pili or fimbriae. These structures play crucial roles in bacterial adhesion, colonization, and biofilm formation, contributing significantly to pathogenicity [107,108].

Bacterial secretion systems are distributed based on cell envelope architecture. Gram-negative bacteria, with both inner and outer membranes, utilize complex systems such as T3SS, T6SS, T2SS, and T5SS, which are absent in Gram-positive species. In contrast, Gram-positive bacteria, which lack an outer membrane, employ systems like the Type VII Secretion System (T7SS) and sortase-dependent mechanisms. Some systems, including the Type IV Secretion System (T4SS) and the Sec/Tat pathways, are conserved across both groups [79,93,97,109,110].

Together, these systems constitute a diverse toolkit used by bacteria for host interactions, environmental adaptation, and biotechnological applications.

While bacterial secretion systems are naturally employed for various physiological functions, their repurposing for therapeutic applications often necessitates the high-level expression of non-native proteins. This overexpression can surpass the secretion system’s inherent capacity, leading to challenges such as protein misfolding, activation of stress responses, and competition for cellular resources, thereby imposing a measurable metabolic burden on the host. For instance, the overproduction of recombinant proteins in *Escherichia coli* has been associated with reduced growth rates and altered metabolism due to the strain on the protein folding and secretion machinery [81]. Additionally, the accumulation of misfolded proteins can trigger stress responses, further impacting cellular homeostasis [123].

In synthetic biology applications, enhancing secretion efficiency and minimizing cellular stress are key design considerations. Signal peptides (SPs) play a critical role in directing therapeutic proteins through bacterial secretion pathways, and their optimization has proven essential for improving secretion yields. Empirical screening of SP libraries, such as the systematic evaluation of over 170 native SPs from *Bacillus subtilis*, has shown significant variability in secretion efficiency, underscoring the importance of matching SPs to specific cargo proteins [124]. Further refinements through rational design or site-directed mutagenesis of SP regions been used to increase protein transport efficiency across membranes [125,126]. In parallel, synthetic gene circuits have been developed to reduce the metabolic burden imposed by high-level expression of recombinant proteins. Strategies such as resource-aware circuit design, orthogonal ribosome usage, and implementation of incoherent feedforward loops (iFFLs) have enabled decoupling of synthetic module expression from host growth processes, preserving bacterial fitness while maintaining production levels [127–130]. Together, these approaches offer robust solutions to improve protein secretion efficiency and system stability and are critical for optimizing secretion-based bacterial therapeutics.

### 3.2. Bacterial surface display

Bacterial surface display is an advanced technique in biotechnology and molecular biology that enables the presentation of proteins or peptides on the surface of bacterial cells, with *E. coli* serving as the primary platform for this technology [131]. Over the years, this approach has gained increasing attention due to its diverse applications in areas such as vaccine development [132,133], peptide library screening [134], and catalysis [135]. The advantages of bacterial surface display include its ease of genetic manipulation, cost-effectiveness compared to mammalian cell systems, and the ability to rapidly screen large libraries of peptides or proteins—capabilities that are invaluable in drug development and protein engineering.

The origins of bacterial surface display can be traced back to the 1980s, a period marked by significant advances in genetic engineering and recombinant DNA technology. During this time, scientists began to unravel the mechanisms of protein targeting and export in bacteria, laying the groundwork for the development of more sophisticated applications like protein display. The earliest examples of bacterial surface display emerged in the late 1980s to early 1990s, when researchers successfully fused foreign proteins or peptides to bacterial surface proteins. Notably, one of the first systems utilized was the fusion of proteins to the outer membrane protein (OMP) LamB of *E. coli*, which demonstrated that foreign proteins could be displayed on bacterial surfaces while maintaining their functionality [136]. In the late 1990s, the application of bacterial surface display expanded to include fields such as vaccine development, where surface-displayed antigens could directly stimulate immune responses, and bio-catalysis, leveraging enzymes displayed on bacterial surfaces for industrial processes [131,137]. The 2000s witnessed further advancements, with the integration of high-throughput screening techniques that facilitated the rapid analysis and optimization of displayed proteins [134,138]. Recent innovations have focused on improving the stability and expression levels of surface-displayed proteins and broadening the range of potential applications [139,140]. To date, bacterial surface display has become a vital tool in areas such as immune engineering, biosensor development, and environmental biotechnology [141–143].

While protein surface display on bacteria is achieved by anchoring foreign proteins to the bacterial cell envelope, the mechanisms differ between Gram-negative and Gram-positive bacteria due to their unique cell envelope structures [144,145]. We will discuss the different mechanisms and therapeutic applications depending on Gram-negative versus Gram-positive bacteria.

#### 3.2.1. Biological mechanisms of bacterial surface display in gram-negative bacteria

In Gram-negative bacteria, surface display of proteins depends on the cellular machinery responsible for the biogenesis and integration of outer membrane proteins (OMPs). The bacterial envelope consists of an inner (cytoplasmic) membrane, a periplasmic space containing a thin peptidoglycan layer, and an asymmetric outer membrane (OM) enriched with β-barrel OMPs. These β-barrels are essential for nutrient uptake, signal transduction, adhesion, and, notably, surface display technologies [146,147]. Surface display in Gram-negative bacteria relies on the localization of heterologous proteins to the outer membrane, typically by fusion to native outer membrane proteins (OMPs), autotransporters, or other β-barrel structures capable of surface exposure [146,147]. The display protein or domain of interest is genetically fused to an anchoring scaffold, commonly an OMP such as OmpA or an autotransporter like AIDA-I, which possesses its own N-terminal signal peptide for translocation through the Sec pathway. After Sec-dependent transport into the periplasm, these anchoring proteins are inserted into the outer membrane, frequently with the assistance of the β-barrel assembly machinery (Bam complex), which recognizes and facilitates integration of β-barrel domains. The surface-exposed domain is then displayed on the bacterial surface, tethered via the outer membrane anchor. This multi-step process ensures correct membrane localization, folding, and surface exposure, enabling applications such as antigen presentation, biosensing, or adhesion. Clarifying this mechanism highlights that the fusion partner, not the passenger protein alone, dictates membrane targeting and surface exposure, thereby guiding effective design of display constructs [101,148].

In Gram-negative bacteria, a diverse array of surface display systems has evolved to facilitate the localization of proteins on the cell surface, with each system offering distinct mechanistic advantages for biotechnological and pathogenic functions. Among these, autotransporters (Type Va secretion systems) are the most extensively characterized. These proteins consist of an N-terminal passenger domain, which harbors the functional activity (e.g., enzymatic or adhesive properties), and a C-terminal β-barrel translocator domain that integrates into the outer membrane (OM) and facilitates the translocation of the passenger to the cell surface. In this context, surface-displayed proteins are typically engineered by replacing the catalytic passenger domain with a protein of interest, which remains tethered to the β-barrel translocator domain that inserts into the outer membrane. This β-barrel facilitates the translocation of the fused protein across the outer membrane. Afterwards, some passenger domains remain tethered (e.g., Antigen 43 in *E. coli*, Hap in *Haemophilus influenzae*), while others, such as the IgA1 protease in *Neisseria* or SepA in *Shigella flexneri*, are released into the extracellular environment after cleavage [100,149,150].

In addition to secretion systems, proteins can be stably localized to the bacterial surface through lipoprotein anchoring, wherein lipid-modified signal peptides direct covalent attachment of proteins to the inner leaflet of the OM. A recent study by Romero-Orejon et al. (2024) exemplifies this strategy by engineering *E. coli* to display PET-degrading enzymes on the surface via fusion to the Braun’s lipoprotein (Lpp) signal peptide. This N-terminal lipid modification enabled covalent anchoring of PETases to the OM, resulting in effective surface exposure and functional degradation of PET substrates [155]. This application highlights the potential of lipoprotein anchoring, such as Lpp and Lgt-fusion constructs, as compact and effective scaffolds for bacterial surface engineering [155–157]. Finally, pilus and fimbrial systems use the chaperone-usher pathway, in which pilin subunits are guided through the periplasm by chaperones and assembled into functional surface structures at the OM via usher proteins. This strategy can be harnessed for surface display of heterologous domains, as demonstrated with type 1 pili (FimA), P pili (PapA), and curli fibers [158–162]. Together, these systems provide powerful and versatile platforms for bacterial surface engineering, with broad applications in vaccine development, biosensing, and synthetic biology.

#### 3.2.2. Existing bacterial surface display systems in gram-negative bacteria

Recent advancements have demonstrated the potential of bacterial surface display systems in therapeutic applications. The synergy between tumor-targeting bacteria and surface-displayed therapeutic proteins provides multiple advantages that enhance the efficacy and precision of cancer therapies. First, bacteria such as *E. coli* and *Salmonella* preferentially colonize the hypoxic and immune-privileged regions of tumors, offering inherent tumor tropism that enhances localized delivery of therapeutics [163,164]. This active targeting is further enhanced by surface-displayed proteins, such as binding peptides, nanobodies or single chain variable fragments, which facilitate tighter binding to tumor-specific antigens and improve localization [165–168]. Second, their active motility and chemotaxis allow bacteria to penetrate deep into poorly vascularized tumor tissues, overcoming interstitial barriers that limit traditional drug penetration, which could potentially be enhanced by overexpressing mobility mode on the surface of bacteria [169,170]. Third, localized expression of immunomodulatory proteins anchored on the surface of bacteria within the tumor microenvironment reduces protein diffusion thus subsequent systemic exposure, thereby minimizing off-target immune activation and cytokine-associated toxicities [74]. Lastly, bacterial surface display systems, such as the Lpp-OmpA scaffold, enable stable and high-density presentation of therapeutic proteins directly on the bacterial outer membrane, improving their retention, activity, and durability in situ [171]. These combined attributes support the development of programmable bacterial systems capable of precise, durable, and locally confined immunotherapy.

**Fig. 4.**
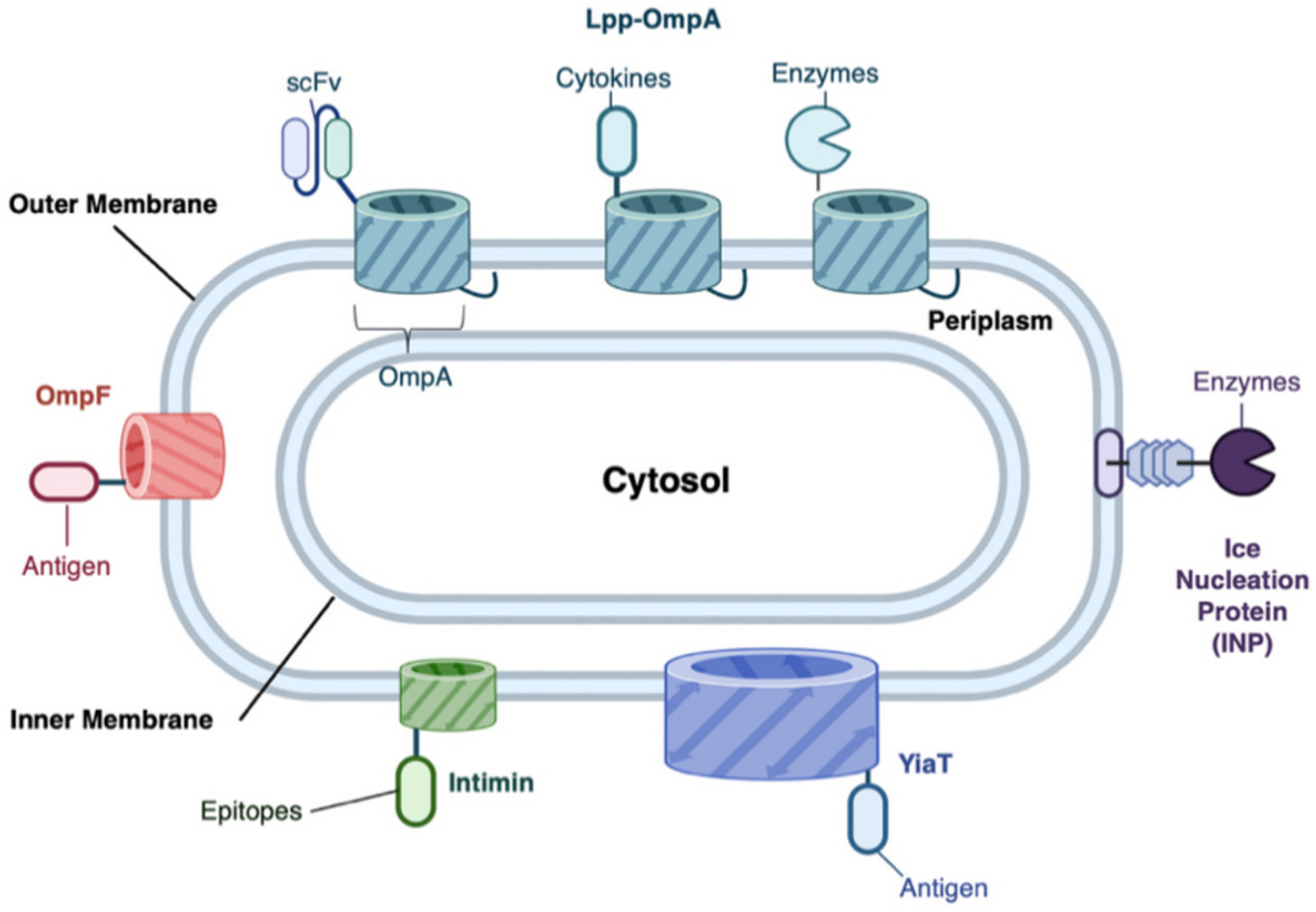
Gram-negative bacterial surface display systems. Lpp-OmpA [143,151,152] is one of the most popular display systems for *E. coli* being used to display scFv antibody fragments, cytokines, and enzymes to create live biotherapeutics. Ice Nucleation Protein (INP) is commonly used to display enzymes such as organophosphorus hydrolase and laccase for biosensors. YiaT [153] and OmpF [154] are commonly used to display antigens and other cytokines for immunotherapies. The cartoons were generated using BioRender.

Existing bacterial surface display systems in Gram-negative bacteria utilize a variety of anchoring mechanisms to present foreign proteins on the cell surface effectively (Fig. 4), and we have categorized different anchor proteins and the associated protein cargos into *E. coli*-based and non-*E. coli* Gram-negative systems (Tables 1 and 2).

**Fig. 5.**
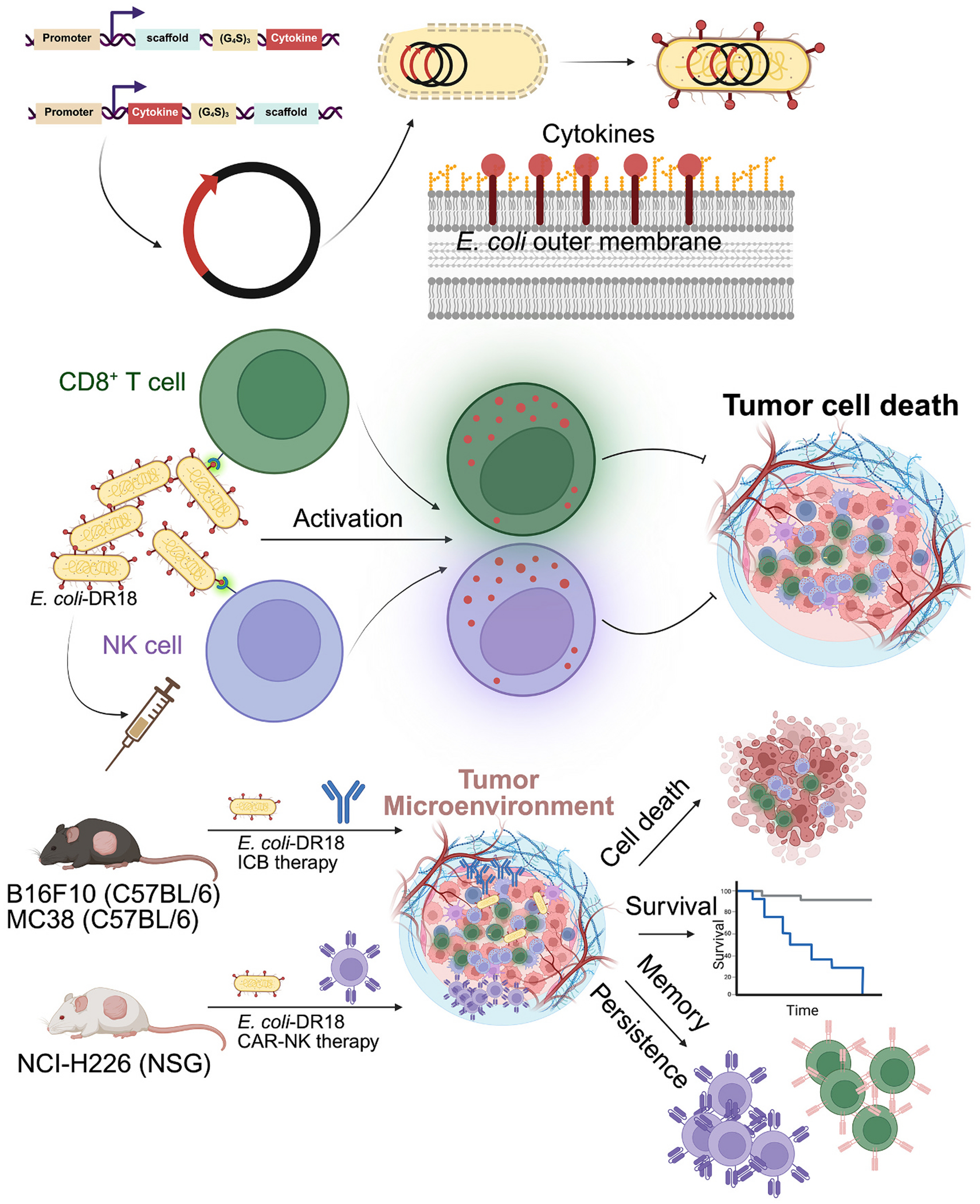
Decoy resistant IL18 (mDR-18) displayed by a bacterial outer membrane protein induces potent anti-tumor responses. Schematic representation of the approach to surface display murine and human cytokines in non-pathogenic *E. coli* as a promising platform for immunotherapy, with *E. coli* displaying decoy-resistant IL18 mutein (DR18) being most effective in vitro and in immune-competent MC38 and B16F10 models and NSG mice bearing mesothelioma tumor cells treated with CAR NK cells. Figures were adapted from *Yang* et al., *Nature Biotechnology*, 2024 [171].

In *E. coli*, popular systems include the use of Lpp-OmpA (Braun’s Lipoprotein-Outer membrane protein A) fusion. A notable example involves the engineering of non-pathogenic *E. coli* to display a murine decoy-resistant IL18 mutein using the Lpp-OmpA anchoring system, which significantly enhanced anti-tumor immunity by activating NK and T cells. This strategy led to improved tumor suppression in preclinical models, highlighting the versatility of surface-displayed cytokines in cancer immunotherapy (Fig. 5) [171]. Additionally, the display of ClbS, a colibactin-neutralizing protein, on the surface of *E. coli* via the Lpp-OmpA system has shown efficacy in neutralizing the genotoxic effects of pathogenic gut bacteria directly in the gut environment. This approach prevented DNA damage and tumorigenesis in murine models, suggesting a novel preventive strategy against *pks*^+^ bacterial strains associated with colorectal cancer [173]. These studies underscore the potential of leveraging bacterial surface display systems for therapeutic delivery and in situ detoxification of pathogenic factors in the gut microbiome. In addition to Lpp-OmpA fusions, Ice nucleation protein (INP)-based systems enable the display of enzymes like organophosphorus hydrolase and laccase for biosensor applications. Additionally, putative outer membrane protein YiaT and OmpF (outer membrane protein F) anchoring systems have been explored for presenting antigens for immunotherapies and vaccine development, such as human decoy resistant IL18 [171] and epitopes of the Hepatitis B virus [154]. In non-*E. coli* Gram-negative bacteria, systems such as OmpA in *Salmonella typhimurium* have been applied to display RGD peptides for cancer therapy [167] and B-cell epitopes [182] for vaccine development.

Among different cargoes displayed by Gram-negative bacteria, nanobodies (Nbs) have become an increasingly popular class of molecules for therapeutic delivery and targeting. Nbs offer significant advantages in therapeutics compared to traditional monoclonal antibodies due to their smaller size (~15 kDa, 4 nm, and 2.5 nm wide) and high structural integrity. While Nbs and traditional antibodies have distinct strength and limitations, their applications depend on specific therapeutic goals. Composed of camelid heavy-chain variable domains (VHH), Nbs consist of four conserved framework regions, and three hypervariable complementary regions, enabling enhanced binding in areas inaccessible to larger antibodies. Their low immunogenicity allows for diverse administration routes; oral, intraperitoneal, and cell surface expression [187,188]. The small size of Nbs allows accessible tissue penetration and fast clearance, making them ideal for specific cancer targeting and whole-body imaging [188]. However, Nbs have a shorter half-life compared to traditional antibodies, necessitating the need for frequent dosing in therapeutic applications. When selecting between Nbs and antibodies for therapeutic use, the decision should be based upon desired outcome [189,190]. For example, Nbs are ideal for deep penetration and targeting of specific cancer or tissues. Some common genera of obligate and facultative anaerobic bacteria such as *Escherichia, Salmonella*, and *Clostridium* expressing surface nanobodies could significantly enhance cancer specific targeting [165,191]. For instance, the probiotic strain *EcN* has been engineered to display Nbs targeting calreticulin (CALR). CALR, typically an ER-resident multifunctional protein involved in protein quality control, can translocate to the cell surface in response to certain cancer therapies or ER stress. This translocation has been observed in various cancer types including esophageal carcinoma, pancreatic adenocarcinoma, prostate adenocarcinoma, and gastric adenocarcinoma [187]. By utilizing *EcN* displaying anti-CALR Nbs, researchers aim to combine the natural anti-tumor effects of *E. coli* with targeted Nb therapy against CALR-expressing cancers for improving treatment efficiency [191].

Additionally, Nbs can be displayed on the surface of bacteria to function as cell-cell adhesion molecules (CAMs), through engineered adhesion toolboxes. CAMs are typically transmembrane proteins that facilitate cell-to-cell or cell-to-extracellular matrix interactions. This property can be leveraged to control cell-cell morphology and specifically target cells displaying the antigen or protein of interest [192]. The Nb-ag tools have shown to be orthogonal and composable through aggregation assays, where two different strains displaying partnered and unpartnered Nb-ag are mixed. These assays demonstrate aggregation when partnered Nb-ag are mixed and no aggregation when unpartnered Nb-ag are mixed. Previous studies have shown that 97.7 % of Nb and antigen pairings self-aggregate when mixed with specific Nb-ag pairings [192].

**Fig. 6.**
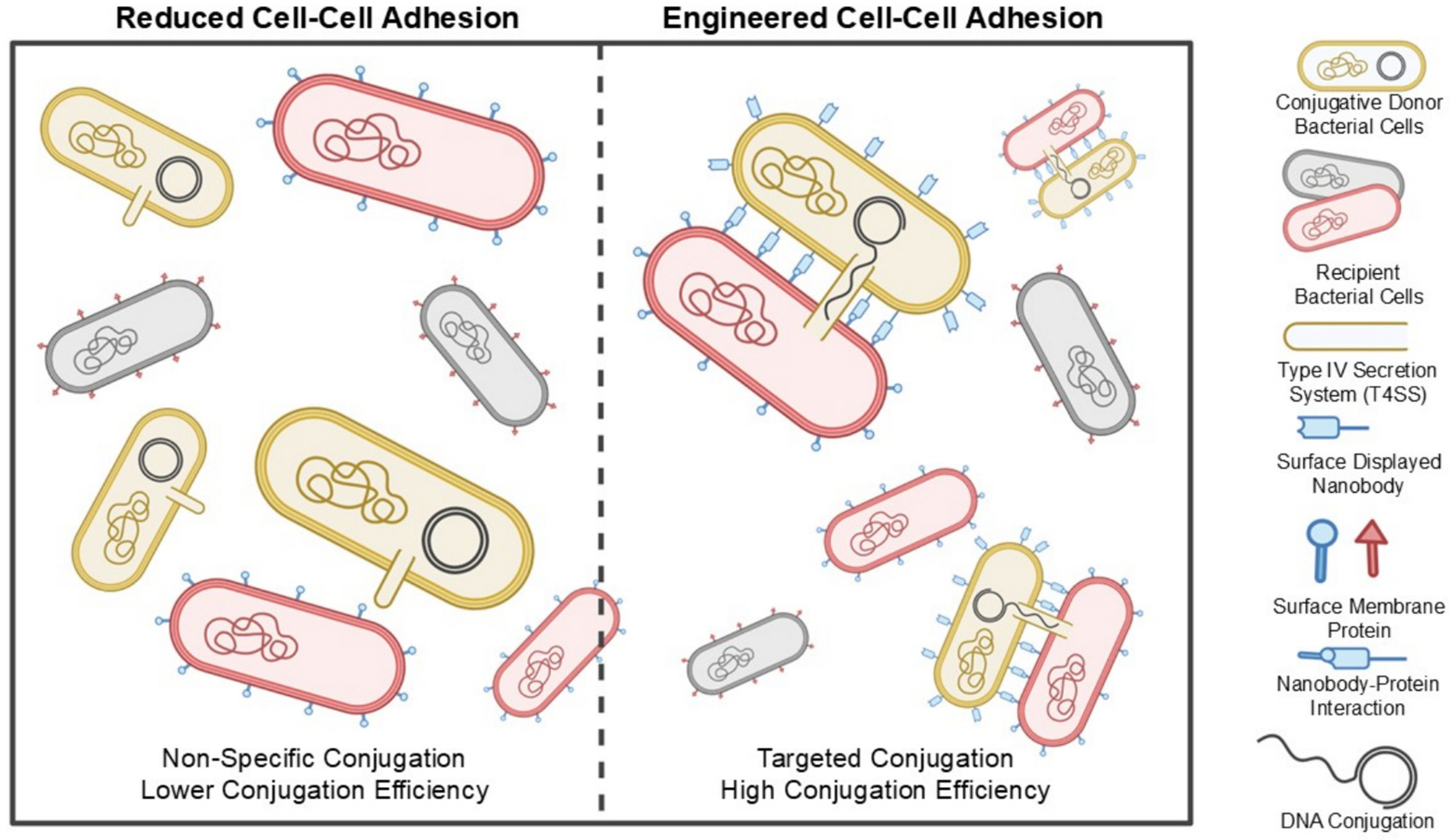
Engineering Precise Plasmid Conjugation between specific donor and recipient cells. Donor cells containing conjugative plasmids rely on conjugative systems T4SSs to maintain mating with recipient strains. The T4SS establishes cell-cell contact and mediates DNA transfer. Engineering donor cells to display either antigens or nanobodies (Nbs) on their surface enhances binding to recipient cells, thereby facilitating increased conjugation efficiency. Image adapted from Ref. [193]

In addition to controlling synthetic bacterial interactions, displaying Nbs on the outer membrane of Gram-negative bacteria can facilitate conjugation and bacterial colonization by targeting a variety of different surface displayed proteins that are naturally present on the native bacteria found in the human body [76,193,194]. Of particular interest, OmpA, one of the most abundant surface displayed proteins on *E. coli* and other *Enterobacteriaceae*, is a multifunctional protein with roles in structural integrity and bacterial plasmid conjugation, and it also serves as a target in host-cell immune defense during infections [195]. Recent discoveries have led to the development of Nbs acting as CAMs that target TraN (outer membrane protein encoded by Fertility plasmid critical for bacterial conjugation in *E. coli* and related bacteria) [196], OmpA and OmpC which can now be engineered with bacterial strains for future applications and therapeutics (Fig. 6) [193]. To enhance conjugation efficiency, researchers have explored the combination of Nbs and bacterial conjugative systems. Specifically, utilizing T4SS and Nbs targeting OmpA expressed on the surface of *E. coli* strains would improve binding to native human bacterial strains expressing OmpA [193]. This approach optimizes the efficiency of delivery conjugative plasmids carrying the genes to the protein of interest to native microbiota [193,197,198].

#### 3.2.3. Future recommendations for integrating bioinformatic prediction and AlphaFold to discover less characterized OMPs

**Fig. 7.**
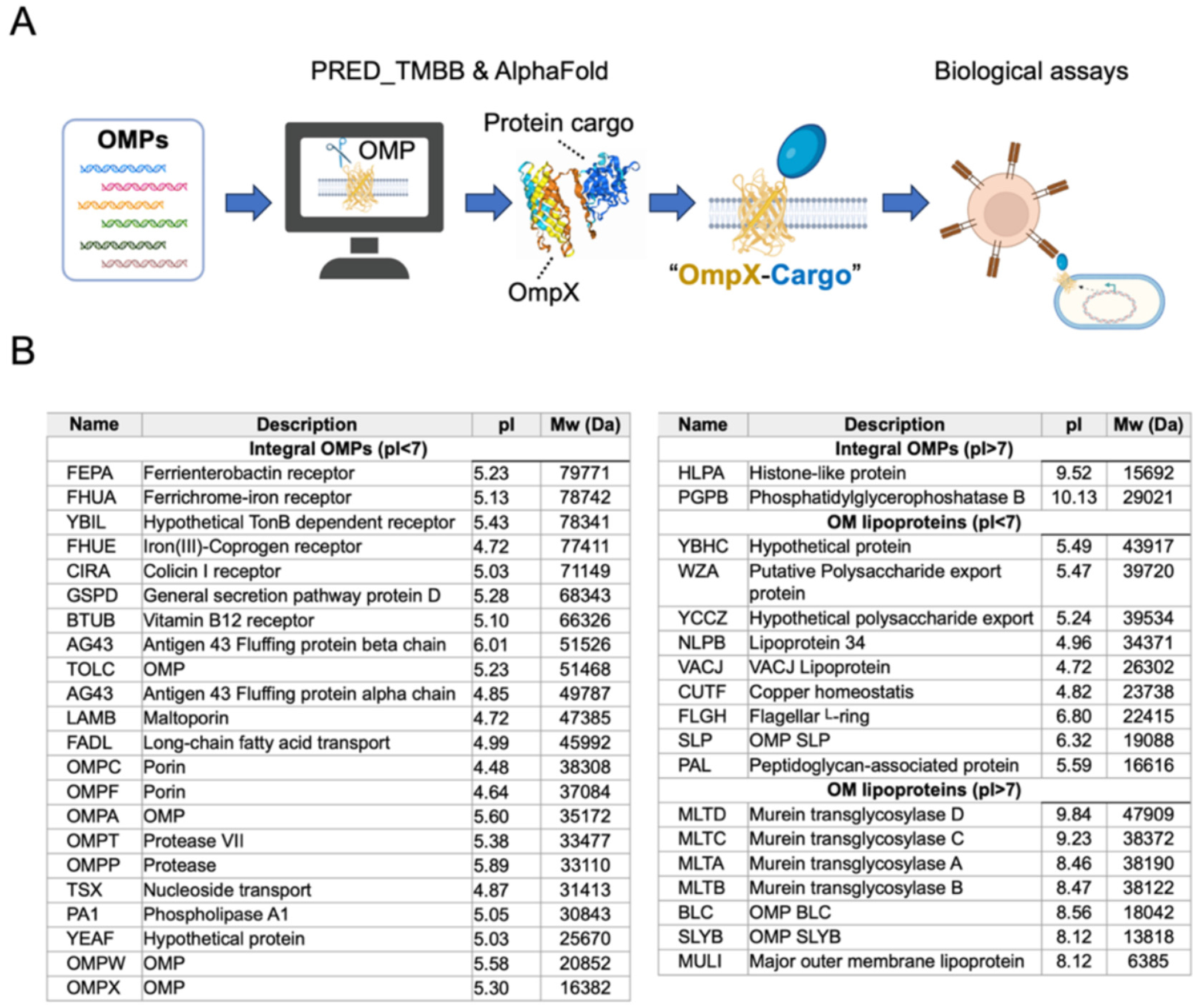
Future recommendations for integrating bioinformatic prediction and AlphaFold to discover less-characterized OMPs. **(A)** Enhance the display efficiency and functional activities of protein cargos via identifying a lead OMP (referred to as “OmpX”) in *E. coli* K-12. The cartoons were generated using BioRender. **(B)** A library of ~40 well-characterized OMPs in *E. coli* [199].

Numerous examples of bacterial surface display in Gram-negative bacteria have been investigated for diverse applications (Tables 1 and 2). However, only a handful of OMPs have been explored for the functional display of heterologous proteins on the surface of *E. coli*, and most of them have been identified through iterative trial-and-error efforts [131,178,192,200]. To enable rapid and rational discovery of optimal OMPs (referred to as “OmpX”) for displaying therapeutic proteins, we reason that integrating computational and experimental tools can significantly accelerate this process (Fig. 7A). For example, a promising approach can be screening approximately 40 known OMPs with diverse characteristics from *E. coli* K12 MG1655 [199] (Fig. 7B), which are categorized into four subfamilies according to their subcellular location and physical properties: (1) integral OMPs, pI 4-7, (2) integral OMPs, pI >7, (3) OM lipoproteins, pI 4-7, and (4) OM lipoproteins, pI >7. To systematically evaluate these OMPs, we can utilize PRED-TMBB [201], a server designed for analyzing the topology of bacterial OMPs, to predict the sequences of extracellular loops in selected OMPs. In cases where a candidate OMP lacks an experimentally determined 3D structure, we envision that AlphaFold, an AI-based protein structure prediction tool, can be employed to predict both the topology and the extracellular loops of these OMPs [202]. The structural insights provided by AlphaFold can ensure that the biologically active site a candidate protein cargo is positioned optimally to avoid steric hindrance and enhance functional display on the bacterial surface.

Notably, although all the lipoproteins listed in Fig. 7B are associated with the outer membrane, several are covalently anchored to its inner leaflet, rendering them unsuitable as scaffolds for surface display due to the absence of extracellularly exposed domains. For example, BLC and PAL are well-characterized lipoproteins that localize to the periplasmic face of the outer membrane without traversing it or presenting surface-accessible epitopes [203,204]. Furthermore, for the majority of outer membrane lipoproteins, the presence and accessibility of extracellular domains remain poorly characterized and require further experimental or computational validation before they can be considered viable candidates for surface display applications.

In summary, integrating AI-driven predictions into a hybrid in silico screening pipeline can streamline the selection of optimal OMPs. Combining structural predictions from AlphaFold with machine learning-based functional predictions makes it possible to focus experimental validation efforts on the most promising candidates. This approach minimizes extensive trial-and-error screening, making the discovery process more efficient and cost-effective. Additionally, by feeding experimental data, such as expression levels and functional assays, back into the machine learning models, the predictive accuracy can be continuously refined, creating a feedback loop that enhances the efficiency of OMP discovery and optimization.

#### 3.2.4. Biological mechanisms and existing bacterial surface display systems in gram-positive bacteria

**Fig. 8.**
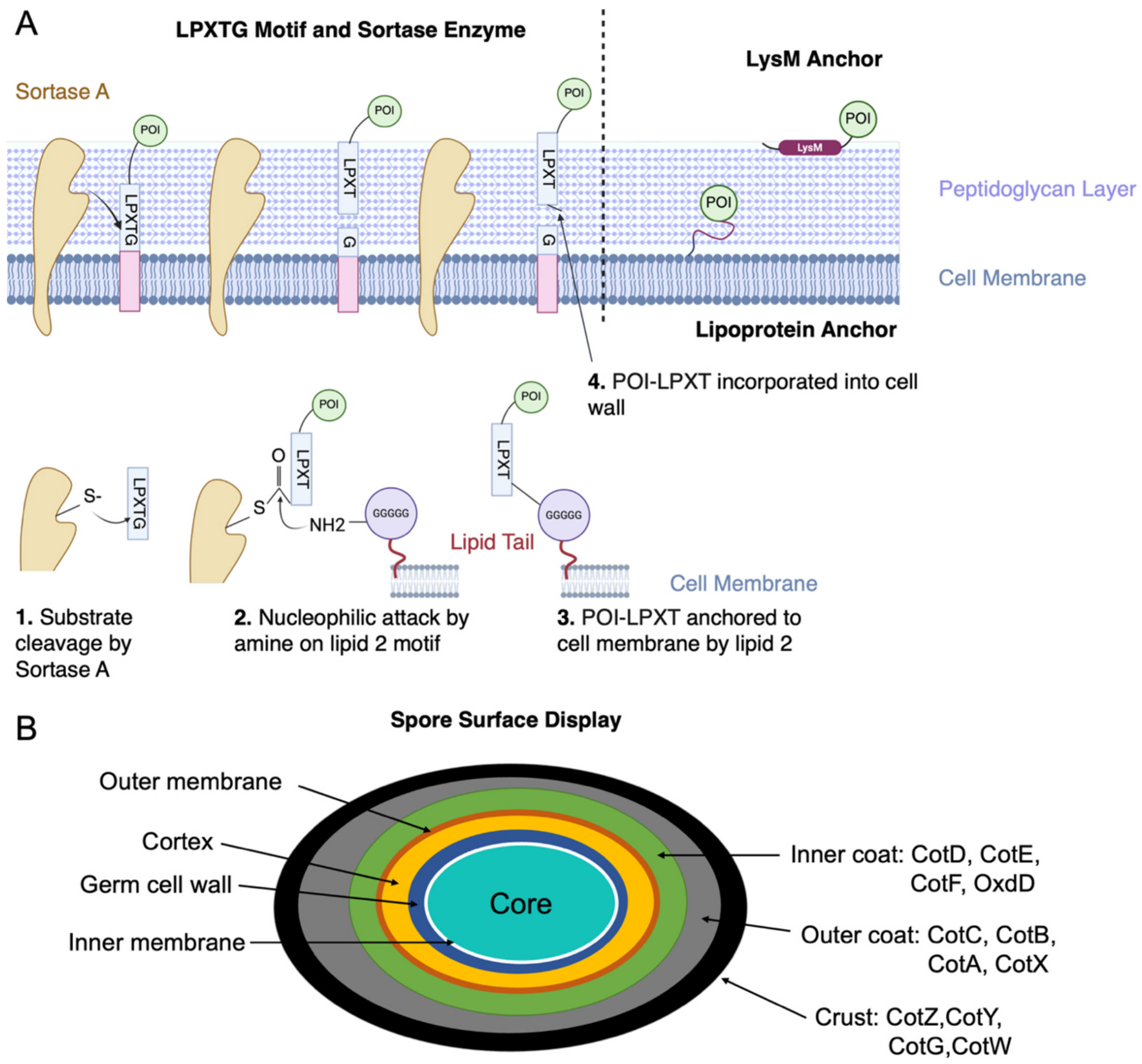
**(A)** Harnessing a sortase enzyme to anchor a protein of interest (POI) containing a LPXTG motif is a common method for surface display on Gram-positive bacteria. Sortase A cleaves between threonine (T) and glycine (G) in the LPTXG motif. This creates an acyl-Sortase A intermediate that is subject to a nucleophilic attack by Lipid II on the cell membrane, through Lipid II’s pentaglycine (GGGGG) [205]. The LPXT and POI is then anchored to the cell membrane through an amide bond. Subsequently, transglycosylation and transpeptidation result in incorporation of POI-LPXT into the cell wall [211,212]. **(B)** A schematic of *B. subtilis* spore structure containing spore-specific proteins for the spore display. These proteins can be in the inner coat, outer coat, and crust of the spore. The cartoons were generated using BioRender.

Gram-positive bacteria have a simpler cell envelope, with a single cytoplasmic membrane surrounded by a thick peptidoglycan layer. This structure allows for different anchoring methods to achieve surface display, including but not limited to LPXTG motif coupled with the use sortase enzyme and the spore surface display (Fig. 8, Tables 3 and 4). In many Gram-positive bacteria, proteins are displayed on the cell surface through the action of sortase enzymes [205,206]. The protein of interest is genetically fused to an LPXTG motif, which is recognized by the sortase enzyme. The sortase cleaves the protein at the LPXTG site and covalently attaches it to the peptidoglycan layer. This method provides a stable display and is commonly used for displaying antigens or enzymes. Additionally, proteins can also be anchored to the cell wall using anchor peptides that interact non-covalently with the peptidoglycan layer. This method allows for flexible and reversible display, useful for applications where temporary interaction with the target is required [207–210]. (See Table 5.)

In terms of the spore surface display, some Gram-positive bacteria, such as *B. subtilis*, can form spores that display proteins on their surface. Proteins are fused to spore coat proteins, allowing them to be stably presented on the spore surface. This method is particularly useful for vaccine applications because spores are resistant to harsh environmental conditions, making them ideal for oral delivery [95,96,168–171].

By genetically engineering bacteria to express antigens or antibodies on their surfaces, researchers can leverage the natural immunostimulatory properties of bacterial cell walls to induce strong immune responses. This approach has been widely used in designing mucosal vaccines, where surface-displayed antigens target immune cells and provoke both humoral and cellular immunity. Notably, *B. subtilis* spores displaying tetanus toxin fragments have been shown to stimulate immune responses in animal models, paving the way for noninvasive oral vaccines. Additionally, surface display can be adapted to attach nanoliposomes loaded with chemotherapeutic drugs, as demonstrated with *Magnetococcus marinus*, which targets colorectal tumors [213,214]. In this review, *B. subtilis* based spore surface display system will be mainly discussed here.

#### 3.2.5. B. subtilis-based spore display system

*B. subtilis* is a Gram-positive, rod-shaped, endospore-forming bacterium isolated from soil, widely utilized for heterologous protein production. It is generally recognized as safe (GRAS) standards set by the Food and Drug Administration (FDA), which provide a long track record of safety and efficacy in human probiotic supplements [241]. In addition to exploring the vegetative cells to produce recombinant proteins, *B. subtilis* could form endospores, which can tolerate harsh environments such as extreme pH, UV, sonication, etc. [242] The history for using *B. subtilis* spore surface display system could be traced back to 2001 developed by Isticato et al., who used the *B. subtilis* spore coat protein CotB to display the C-terminal fragment of the tetanus toxin (TTFC) on the spore surface [243]. This system was shown to be effective in inducing an immune response in mice, and it paved the way for the development of other spore surface display systems. Since then, spore surface display has been used to display a variety of proteins, including enzymes, antigens, and antibodies [244,245]. Researchers have also explored using spores for various applications, including bioremediation and the generation and screening of mutagenesis libraries [246,247].

##### 3.2.5.1. The structure of spore.

*B. subtilis* spores have a unique multilayer structure contributing to their resilience and suitability for surface display [248–250]. The spore has several layers, including the core, cortex, and coat [251].

The core contains genetic material and essential enzymes such as DNA, RNA, ribosomes, and dipicolinic acid (DPA) complexed with calcium, stabilizing macromolecules and enhancing heat resistance [252]. The inner membrane surrounds the core, acting as a selective barrier enclosed by germ cell wall [253]. Encapsulated by an outer membrane, the cortex, a thick layer of modified peptidoglycan, provides crucial resistance to osmotic stress [251]. Anchored to this outer membrane are SpoIVA and SpoVM proteins, forming the foundational basement layer of the spore coat, a multilayered protein shell, acting as a formidable barrier, shielding the spore from harsh environmental conditions [254]. Progressing outwards from the basement layer, the coat is organized into distinct strata: the inner coat, rich in proteins like CotD, CotE, and SafA; the outer coat, featuring proteins such as CotB, CotC, and CotG; and finally, the outermost layer, the crust, composed of proteins including CotX, CotY, and CotZ [255–257]. The spore coat is particularly interesting for surface display because it comprises various Cot proteins that can be used as anchoring motifs for heterologous proteins [258]. These coat proteins are expressed during sporulation and assembled into a complex structure surrounding the spore. Spore display systems generally refer to the use of genetic engineering methods to immobilize target proteins or small molecules on the surface of spores [259]. In *B. subtilis*, two principal strategies have been investigated: genetic recombinant and non-recombinant methods.

##### 3.2.5.2. Recombinant method-based spore display system.

A well-designed spore surface display system typically has three key design components: 1) choosing appropriate anchor proteins, 2) optimized target proteins, and 3) flexible linkers. These components work together to ensure the successful display of the target protein on the spore surface [260].

###### Anchor proteins:

Anchor proteins are usually the coat proteins that are crucial for attaching the target protein to the spore surface. Appropriate anchor proteins enhance target protein display efficiency and stability by optimizing abundance, location, and compatibility [243–245]. CotB was the first anchor protein identified and used in surface display [243]. To date, various spore coat proteins, such as CotB, CotC, CotE, CotG, CotX, CotY, CotZ, CgeA, and OxdD, have been successfully used to display exogenous proteins on the spore surface [258].

###### Target protein:

Target proteins, which vary depending on the application and can include enzymes, antigens, antibodies, or receptors, are linked to anchor proteins [258]. Typically, target protein sequences are codon-optimized for *B. subtilis*. Additionally, researchers could introduce mutations to achieve specific functionalities through the protein engineering method. Site-directed mutagenesis, for example, allows for the precise substitution, insertion, or deletion of specific amino acids to modify characteristics such as structural stability, secondary structure, solubility, and activity etc. [261] Alternatively, researchers could also use random mutagenesis to generate a library of protein variants, which can then be screened to identify recombinant proteins exhibiting desired functions [262].

###### Linkers:

Linkers are usually 5 to 10 repeats of aliphatic amino acids like glycine and serine which are used to connect the anchor protein and the target protein [258,263]. They enhance display efficiency and stability by providing flexibility and spacing, reducing steric hindrance [264,265], and facilitating proper folding and activity. (GGGGS)n, (SGGS)n, and a 10-alanine linker are commonly used linkers [266]. Besides this, there are also cleavable linkers, functional linkers, and rigid linkers which depends on the application of the system [266,267]. Recently, Cohesin-dockerin (Coh-Doc) modules are newly explored ‘linker’ [268]. Coh-Doc are protein domains that mediate high-affinity interactions between proteins. It was originally found in cellulosomes which are produced by bacteria to degrade cellulose [269]. In the spore display system, the cohesin module is typically fused to the anchor protein, and the dockerin module is fused to the target protein. The interaction between the cohesin and dockerin modules brings the target protein close to the spore surface, increasing the display efficiency [270]. Until now, different Coh-Doc modules have been used in spore surface display, including type I, type II, and type III modules [269].

##### 3.2.5.3. Non-recombinant method based spore display system.

Besides the recombinant method, scientists have also developed non-recombinant method to direct absorb of target proteins onto the exterior surface of *Bacillus subtilis* of spores. It relies on a combination of electrostatic forces, hydrogen bonding, and hydrophobic interactions to passively adsorb the target protein to bypass the need for genetic engineering [271]. The surface of *B. subtilis* spores is negatively charged and hydrophobic, which means that target proteins should ideally be positively charged and hydrophobic [272–274]. This method provides several advantages. First, it is fast and easy to operate, avoiding complex design and time-consuming genetic engineering steps. Second, the final spore product is not considered a genetically modified material, reducing safety concerns for regulatory aspects. Third, this method can work well for specific proteins by achieving higher display levels, requiring fewer spores, or maintaining the native protein configuration [275]. However, with this method, it is difficult to control the total amount of protein adsorbed onto the surface, making it challenging to achieve a stable display density [275]. Ultimately, this method relies purely on the combination of physical interactions, which means the results can be significantly influenced by the properties of the displayed proteins, the spore strain [276] and sporulation conditions [259,277].

Researchers have successfully used spores to adsorb the B subunit of *E. coli* heat-labile enterotoxin (LTB) onto their surface to develop an oral vaccine [277]. This process was achieved at pH 4 through a combination of electrostatic and hydrophobic interactions. The most important point is that the LTB was displayed more efficiently via non-recombinant method compared to the traditional recombinant method, and it also required 70 times fewer spores. Besides this, Wu et al. also explored displaying Phenylalanine Ammonia Lyase (PAL) using both non-recombinant and recombinant approaches [278]. Although they didn’t directly compare the efficiency of these two methods, their work illustrated that the non-recombinant method could successfully display a gastrointestinal environment stabled and functional therapeutic enzyme.

#### 3.2.6. Applications of surface display system based on gram-positive strains

*B. subtilis* spore surface display system is a promising technology with the potential to revolutionize animal health applications. It has been successfully used for vaccine development, disease prevention, and improving nutrient utilization in animals through recombinant method.

Compared with traditional vaccine approaches, the spore surface display system has several advantages when developing as the oral vaccine: 1) the robust nature, safety, and ability of *B. subtilis* spores to survive harsh conditions make them ideal for oral administration to elicit an immune response, 2) it allows for the presentation of heterologous antigens in their native form, without the need for fusion proteins which can cause the adverse reactions, 3) the cost-effectiveness of spore production, storage, transport, and feeding makes it attractive for animals.

Studies have shown that spores displaying antigens from bacteria pathogens such as *Actinobacillus pleuropneumoniae*, can induce mucosal and systemic immune responses in mice [279]. Vogt et al. engineered spore surface for displaying TasA as a dog vaccine [280]. They observed that germinated *B. subtilis* spores, upon becoming vegetative recombinant bacteria in the gut, facilitated intestinal biofilm formation, stimulating gut-associated lymphoid tissue (GALT) and eliciting a humoral response against TasA fused to an epitope.

Cysteine protease of *Clonorchis sinensis* (*C. sinensis*) (CsCP) serves as an endogenous key component in the excystment of metacercariae and other physiological and pathological processes. Tang et al. developed a CotC-CsCP spore surface system, which can significantly induce IgG and isotypes expression without causing any inflammatory injury [281]. Besides this, displaying foot-and-mouth disease virus, avian influenza virus, porcine reproductive and respiratory syndrome virus, classical swine fever virus was all showed success in inducing protective immunity in animals.

Spores displaying enzymes or antimicrobial peptides can be used to prevent or treat animal infections. For example, spores displaying chitinase have been shown to inhibit the growth of plant fungi, suggesting their potential as biopesticides [282]. Spores can also be used for bioremediation in animal health, such as removing heavy metals or degrading environmental pollutants. Wróbel et al. developed *B. subtilis* to display metallothionein to bind and remove heavy metals from contaminated environment [283,284]. Regarding improving nutrient utilization, studies showed that displaying phytase can increase the bioavailability of phosphorus from phytate, a common anti-nutritional factor in animal feed [285]. Other than that, displaying enzymes that degrade mycotoxins in animal feed can also improve animal health by reducing the toxic effects of these contaminants to improve animal digestibility [286].

#### 3.2.7. Challenges and future directions

Despite significant achievements in the surface display system, it’s still at very early stage for commercializing. There are still some challenges remaining to be addressed.

The first is choosing an appropriate display method between recombinant and non-recombinant. However, the selection of a particular method is usually determined by several factors. The first is display efficiency and density. The efficacy of the non-recombinant method is variable, depending on the characteristics of the target protein (e.g., size, complexity, and stability), the properties of the spore strain, and the intended application (e.g., vaccine, biocatalyst, biosensor). For example, a CotC-fused protein was expressed at 9.6 × 10^−5^ pg on the spore surface, which is around 0.3 % of the total surface proteins [287]. However, when the same protein was displayed using the non-recombinant method, the total surface amount was reportedly 25 to 70 times higher than with the recombinant method [288]. The recombinant method display levels can be potentially very high but are often limited by protein expression or folding challenges [243,289]. The second consideration is the stability of displayed target protein. For the non-recombinant method, there is a high risk of leaching due to non-covalent interactions [271]. The genetic recombinant method provides display stability due to covalent linkages [263]. The third factor relates to sporulation. The non-recombinant method is typically performed on mature spores and thus has minimal impact on spore [290]. However, the recombinant method can introduce a metabolic burden, which could potentially negatively influence spore viability, sporulation efficiency, or germination process to cause low display efficacy. The final consideration is cost. The non-recombinant method is often a fast and easy process at the bench scale, translating to lower initial costs compared to the recombinant method. However, potential leaching problems with the non-recombinant method may necessitate extensive troubleshooting and optimization steps, which can add to the overall time and expense. Thus, choosing between non-recombinant versus recombinant display systems is a multi-dimensional decision that is highly purpose driven. For example, for high-throughput screening, the non-recombinant method might be considered to save time and reduce costs. When displaying very large proteins that are difficult to fuse or express, using the non-recombinant method may be advantageous. When developing diagnostic tools, the recombinant method may be preferred due to its greater display stability, suitability for long-term storage, and consistent display characteristics. Furthermore, a combination of both methods could also be considered, depending on specific needs.

The second is efficiently expressing target proteins with high yield and low cost when using recombinant method. This requires optimizing various factors, including the vector, anchor protein, linker, and target protein. AI and machine learning technologies offer a promising direction for creating better expression and stable spore systems by developing algorithms that optimize factor combinations, using the analysis of vast protein sequence, structure, and expression level data.

Third, better strategies are needed to ensure the stability and functionality of displayed proteins. This includes addressing potential issues related to protein folding, and degradation. Besides this, combing with other technologies, such as adjuvants or alternative delivery systems could also increase the functionality of target protein.

Third, to commercialize spore surface display system products, it is necessary to develop methods for producing large quantities of spores displaying the target protein at a low cost. Besides this, spores are easy to cause contamination during the production process, so a proper production pipeline is needed.

### 3.3. Lysis-based and minicell-based delivery

The lysis-based delivery mechanism involves genetically engineering bacteria to accumulate therapeutic proteins or nucleic acids released upon cell lysis. This strategy can potentially treat cancers and infections where targeted delivery is essential. For example, *Lactobacillus reuteri* has been modified to release IL-22 in response to prophage-induced lysis, which has shown efficacy in treating liver disease and radiation-induced tissue damage in preclinical models [291–294]. Moreover, engineered *EcN* strains equipped with self-lysis circuit based on quorum-sensing mode and lytic protein phiX174 lysis protein E mode achieved success in targeted delivery of anti-cancer reagents in solid tumor microenvironment [39–43,45].

Bacterial minicells are anucleate, non-replicating vesicles produced from aberrant cell division. Their formation is typically achieved by genetic disruption of the minCDE system, which is responsible for proper septum placement during cell division in rod-shaped bacteria such as *E. coli* and *Salmonella* spp. In the absence of a functional Min system, the cell division machinery mislocalizes, leading to the formation of small, DNA-free vesicles at cell poles. These minicells retain functional membranes, ATP stores, ribosomes, secretion systems, and metabolic activity, while being incapable of replication or gene transcription due to the absence of chromosomal DNA [295–297].

Minicells have been developed as delivery vehicles for a range of therapeutics, including chemotherapeutic drugs, nucleic acids, and immunomodulatory proteins. Specifically, minicells can be equipped with surface display proteins for versatile applications [298,299]. One of the most well-documented examples is the use of *S. typhimurium*-derived minicells loaded with doxorubicin or paclitaxel for targeted chemotherapy. These minicells were further conjugated with tumor-targeting antibodies to achieve selective uptake by cancer cells, resulting in significant tumor suppression in xenograft models with minimal systemic toxicity [296,300,301]. Additionally, minicells have been explored as antimicrobial agents combing with surface display technology. For instance, ligand-targeted minicells delivering CRISPR-Cas9 systems have been used to eliminate multidrug-resistant bacteria through type IV secretion systems [76]. The application of minicells for cancer immunotherapy has also been demonstrated, where *Salmonella* minicells were modified to express a functional Type III Secretion System (T3SS), enabling the delivery of antigens directly into the cytosol of antigen-presenting cells (APCs). This direct delivery facilitated robust activation of dendritic cells and elicited strong CD8^+^ T cell responses in murine models, showcasing the potential of minicells as platforms for cancer vaccines [75].

Minicells offer a modular and safe alternative to intact bacteria for clinical applications. Their inability to replicate minimizes the risk of uncontrolled colonization or infection, making them highly suitable for therapeutic use. When combined with advanced protein display technologies and programmable delivery systems, engineered minicells represent a versatile platform for targeted interventions in oncology, infectious diseases, and immunotherapy. Ongoing innovations in minicell engineering, particularly the integration of biosensing circuits and immune evasion modules, are expected to further expand their clinical utility.

## 4. Conclusions

The engineering of bacteria for therapeutic applications represents a frontier in synthetic biology, offering unprecedented versatility and precision in disease treatment and prevention. By harnessing natural bacterial systems, such as secretion, membrane vesicles, surface display, lysis-based and minicell mechanisms, scientists can tailor delivery vehicles to produce, protect, and release therapeutic agents directly at target sites. Each delivery mode provides unique capabilities: secretion systems enable continuous, localized production of biomolecules; membrane vesicles and bacterial ghosts protect and deliver complex cargos; surface display facilitates robust immune activation; and lysis-based systems offer controlled, responsive delivery triggered by environmental cues [214].

The integration of synthetic gene circuits and biosensors has further expanded the potential of these bacterial systems, allowing for precisely timed and localized therapeutic actions in response to specific physiological signals. This programmability holds promise for highly targeted applications in oncology, infectious diseases, and immune modulation, where conventional treatments often lack specificity or efficacy. Nevertheless, the translation of engineered bacteria from laboratory research to clinical application requires addressing several challenges, including achieving stable colonization within host environments, mitigating risks associated with horizontal gene transfer, and navigating ethical and regulatory considerations related to the use of genetically modified organisms in human health.

Ensuring the safety of engineered bacterial therapeutics is paramount, and future efforts must focus on implementing robust containment strategies to prevent unintended spread and impact on the natural microbiome. Advances in gene-editing technologies such as CRISPR-based “kill-switches” [302] or auxotrophic modifications [303], which require engineered bacteria to depend on essential nutrients not found in the body, offer promising solutions for limiting bacterial persistence outside of target environments. Developing methods to program controlled bacterial clearance after therapeutic action will also be crucial for clinical applications, particularly in immunocompromised patients. Comprehensive long-term studies assessing ecological impact, unwanted systemic inflammation, and potential interactions with the native microbiome are essential to ensure that these advanced therapies remain safe and effective.

As the field continues to evolve, interdisciplinary collaboration between microbiologists, bioengineers, clinicians, and immunologists will be essential to ensure the safety and effectiveness of these novel therapies. With continued advances, the programmable and adaptive nature of bacterial therapeutics offers a highly promising pathway toward developing next-generation treatments that are precisely tailored, personalized, and capable of addressing a wide array of complex diseases.

## Figures and Tables

**Table 1:** Representative display systems and applications for *E. coli.*

Anchor	Passenger	Application	Source
Lpp-OmpA	A Single Chain Variable Fragment (scFv)	Peptide and scFv library formulation	[143,151]
Lpp-OmpA	Polyethylene terephthalate	Biocatalysis	[172]
Lpp-OmpA	IL18 mutein (Decoy Resistant IL18)	Immunotherapies	[171]
Lpp-OmpA	Colibactin self-protection protein ClbS	Drug delivery and vaccine development	[173]
INP	Organophosphorus hydrolase	Medical Diagnostics, Biosensors	[174]
INP	Laccase WlacD	Biosensors	[175]
INP	Glutamate dehydro genase (GDH) from *Pandalus borealis*	Biosensors	[176]
VP8 of Rotavirus	Type A histo-blood group antigen (HBGA)	Vaccine development	[177]
YiaT	Cytotoxic T-lymphocyte-associated protein 4 (CTLA4)	Drug delivery and immunotherapies	[178]
OmpF	Epitopes of the Hepatitis B virus (HBV)	Vaccine development	[154]
N-terminus Intimin	Biotinylated antigen	Nanobody production	[179]
PgsA	Cytochrome P450 BM3	Biocatalysis	[180]

**Table 2:** Representative display systems and applications for non-*E. coli* Gram-negative bacteria.

Carrier	Anchor	Passenger	Application	Source
*Pseudomonas putida*	Intimin-β	V_HH_FIB1 nanobody sequence,	Nanobody library formulation	[181]
*Salmonella typhimurium*	OmpA	RGD peptide (ACDCRGDCFCG)	Vaccine development	[167]
*Salmonella typhimurium*	SadA	B-cell epitopes of urease B from *Helicobacter pylori*	Vaccine development	[182]
*Salmonella typhimurium*	INP	domain III of Japanese encephalitis virus E protein	Vaccine development	[183]
*Salmonella enterica*	OmpA	Flagellin protein (FliC)	Immunotherapies	[184]
*Proteus mirabilis*	CCma	Staphylokinase	Vaccine development	[185]
*Klebsiella pneumoniae*	AIDA-I	Nitrilase	Biocatalysis	[186]

**Table 3:** Representative display systems and applications for *B. subtilis*.

Anchor	Passenger	Application	Source
CotB	Tetanus toxin fragment C (TTFC)	Vaccine development against Tetanus	[215]
CotB	Protective antigen (PA)	Vaccine development against *Bacillus anthracis*	[216]
CotC	Paramyosin of *Clonorchis sinensis* (CsPmy)	Vaccine development against *Clonorchis sinensis*	[217]
CotC	Outer membrane protein C	Vaccine development against *Salmonella*	[218]
CotC	Chlorella variabilis Fatty Acid Photodecarboxylase	Biotransformation	[219]
CotC	Baculovirus envelope glycoprotein GP64	Vaccine development against *Bombyx mori* Nucleopolyhedrovirus	[220]
CotC	Human growth hormone	Hormone supplement	[221]
CotC	Anti-CTLA-4 nanobody, anti-PDL-1 nanobody	Nanobody Production	[222]
CotG	Nitrilase	Industrial Biocatalyst	[223]
CotE	Tyrosinase	Industrial Biocatalysis	[224]
YuaB	Repeated histidine-tag	Diagnostics and immunolabeling	[225]
CgeA	Cytotoxin-associated gene A protein (CagA)	Vector system for vaccine development	[226]
CotC	Polymerase acidic protein (PA)	Vaccine development for tuberculosis	[227]
CotY/CotZ	β-galactosidase	Industrial Biocatalysis	[228]
SscA	β-glucuronidase	Industrial Biocatalysis	[229]
CotG	D-Allulose with d-Psicose 3-Epimerase	Industrial Biocatalysis	[230]

**Table 4:** Representative display systems and applications for other Gram-positive bacteria.

Carrier	Anchor	Passenger	Application	Source
*Bacillus coagulans*	CotB	Curcumin and folate, antibody and apclitaxel	Drug Delivery	[231]
*Bacillus thuringiensis*	Mbgn	Laccase WlacD	Vaccine development	[232]
*Staphylococcus carnosus*	ABP	Nanobody Library	Drug delivery and nanobody production	[233]
*Staphylococcus epidermidis*	Sortase signal sequence	2 W peptide	Immunotherapies	[234]
*ligilactobacillus salivarius*	CbpA	OmpA	Vaccine development	[235]
*Lactobacillus acidophilus*	SlpA	Specific Intercellular adhesion molecule-3-Grabbing Non-integrin Related gene 3 (SIGNR3)	Therapies for autoinflammatory diseases	[236]
*Lactococcus lactis*	M6	Tetanus toxin fragment C (TTFC)	Vaccine development	[237]
*Lactococcus lactis*	SlpA	IL-10, IL-27	Therapies for autoinflammatory diseases	[238]
*Lacticaseibacillus casei*	cA	Merozoite Surface Antigen 2 (MSA2)	Immunotherapies	[239]
*Corynebacterium glutamicum*	PorC	β-Glucosidase	Biocatalysis	[240]

**Table 5:** Full name of surface protein corresponding to the abbreviations used in the previous tables.

Abbreviations	Full name
Lpp-OmpA	Braun’s lipoprotein-Outer membrane protein A
INP	Ice nucleation protein
YiaT	Putative outer membrane protein YiaT
OmpF	Outer membrane protein F
OmpA	Outer membrane protein A
sadA	Autotransporter adhesin sadA
CCma	Cytochrome c biogenesis ATP-binding export protein
AIDA-I	Autotransporter adhesin AIDA-I
CotB	Spore coat protein B
CotC	Spore coat protein C
CotG	Spore coat protein G
YuaB	Uncharacterized protein YuaB
CgeA	Spore crust protein CgeA
CotY	Spore coat protein Y
CotZ	Spore coat protein Z
SscA	Small spore coat assembly protein A
Mbgn	N-terminal domain (Mbgn) of a peptidoglycan hydrolase (Mbg)
ABP	Albumin-binding protein
CbpA	Curved DNA binding protein A
SlpA	S-layer protein A
M6	Uncharacterized protein M6
cA	The peptidoglycan-binding domain of lactococcal AcmA protein
PorC	a mechanosensitive ion channel family porin protein

## Data Availability

No data was used for the research described in the article.
